# Adaptive CoCoLasso for High-Dimensional Measurement Error Models

**DOI:** 10.3390/e27020097

**Published:** 2025-01-21

**Authors:** Qin Yu

**Affiliations:** School of Management, University of Science and Technology of China, Hefei 230026, China; yuqin97@mail.ustc.edu.cn

**Keywords:** Adaptive CoCoLasso, measurement error, high-dimensional regression, nearest positive semi-definite projection

## Abstract

A significant portion of theoretical and empirical studies in high-dimensional regression have primarily concentrated on clean datasets. However, in numerous practical scenarios, data are often corrupted by missing values and measurement errors, which cannot be ignored. Despite the substantial progress in high-dimensional regression with contaminated covariates, methods that achieve an effective trade-off among prediction accuracy, feature selection, and computational efficiency remain significantly underexplored. We introduce adaptive convex conditioned Lasso (Adaptive CoCoLasso), offering a new approach that can handle high-dimensional linear models with error-prone measurements. This estimator combines a projection onto the nearest positive semi-definite matrix with an adaptively weighted $\ell_{1}$ penalty. Theoretical guarantees are provided by establishing error bounds for the estimators. The results from the synthetic data analysis indicate that the Adaptive CoCoLasso performs strongly in prediction accuracy and mean squared error, particularly in scenarios involving both additive and multiplicative noise in measurements. While the Adaptive CoCoLasso estimator performs comparably or is slightly outperformed by certain methods, such as Hard, in reducing the number of incorrectly identified covariates, its strength lies in offering a more favorable trade-off between prediction accuracy and sparse modeling.

## 1. Introduction

High-dimensional statistical learning has found extensive applications across diverse fields, including artificial intelligence, genomics, molecular biology, and economics. Numerous effective methods leveraging sparse learning through regularization have been developed to facilitate statistical inference in high-dimensional settings. These methods are well-documented in various studies, such as [1,2,3,4,5,6,7,8,9,10,11,12,13], among others. However, most of the previous research has focused on error-free data. In practice, measurement errors are prevalent in applications such as surveys with missing or inaccurate data due to non-responses, voting systems affected by imprecise instruments or systematic biases, and sensor networks corrupted by communication failures or environmental interference. Challenges involving noisy, incomplete, or corrupted data are frequently encountered. Naively applying methods designed for clean datasets to those affected by measurement errors can lead to inconsistent and imprecise estimates, which in turn result in inaccurate conclusions, particularly in high-dimensional settings. Therefore, developing robust methods for model selection and estimation that explicitly account for measurement errors in high-dimensional problems is of paramount importance.

In recent years, sparse modeling in high-dimensional models with measurement errors has garnered widespread attention. For instance, Ref. [14] proposed minimizing regularized least squares while accounting for additive measurement errors in the covariate matrices of partially linear models. In high-dimensional linear sparse regression, Ref. [15] developed a Lasso-type estimator that utilizes an unbiased approximation to replace the corrupted Gram matrix. However, incorporating measurement error information often leads to non-convex likelihood functions, complicating the solution of the associated optimization problems. To address this challenge, Ref. [16] proposed the nearest positive semi-definite projection matrix as an approximation for the unbiased Gram matrix estimate. Using this matrix as a foundation, they introduced the convex conditioned Lasso (CoCoLasso), which reformulates the objective function as a convex optimization problem to facilitate efficient sparse learning in error-prone high-dimensional linear models.

Although CoCoLasso demonstrates superior computational efficiency due to its convex optimization framework, the use of the $\ell_{1}$ penalty imposes the same level of shrinkage on all coefficients, which can introduce biases. This often results in overfitting by selecting an overly complex model to minimize prediction error [1,12,17]. To address the biases and overfitting introduced by the $\ell_{1}$ penalty, Ref. [18] proposed balanced estimation, which is based on the nearest positive semi-definite matrix and incorporates combined $\ell_{1}$ and concave regularization. Although balanced estimation achieves an ideal trade-off between prediction accuracy and variable selection, it suffers from certain limitations. Firstly, the non-convex nature of the concave regularization results in increased computational complexity, rendering its application in high-dimensional settings difficult. Secondly, the selection of tuning parameters for the concave penalty is often difficult and may lead to suboptimal performance in practice.

To address these issues, we propose Adaptive CoCoLasso, which combines the nearest positive semi-definite projection with an adaptive $\ell_{1}$ penalty. The Adaptive CoCoLasso estimator not only preserves the computational efficiency of convex optimization but also achieves precise estimation and feature selection in the presence of both additive and multiplicative measurement errors. By imposing higher penalties on zero coefficients and lower penalties on nonzero coefficients, the Adaptive CoCoLasso minimizes estimation bias and enhances variable selection accuracy. Furthermore, error bounds for the Adaptive CoCoLasso estimator are established, and a theorem guarantees the consistency of support recovery.

This paper makes two primary contributions. First, we propose the Adaptive CoCoLasso estimator for high-dimensional linear regression models where the design matrix is affected by measurement errors, aiming to ensure precise estimation and accurate variable selection. By applying stronger penalties to zero coefficients and weaker penalties to nonzero coefficients, the method effectively mitigates overfitting when dealing with additive and multiplicative measurement errors. In addition, we establish theoretical guarantees for the proposed method by deriving oracle inequalities for prediction and estimation errors and proving the consistency of support recovery. Extensive simulation studies demonstrate the effectiveness of our approach.

The structure of this paper is as follows. Section 2 outlines the model setup and introduces the proposed Adaptive CoCoLasso estimator. Section 3 presents the theoretical properties, including bounds on oracle estimation errors. Section 4 evaluates the finite-sample performance of the proposed method through simulation studies. We provide all the proofs in the Appendix A.

**Notation 1.**  *For a vector $x={\left(x_{1},\ldots ,x_{p}\right)}^{⊤}$, the $\ell_{q}$ norm is defined as ${∥x∥}_{q}={\left(\sum_{j=1}^{p}{\left|x_{j}\right|}^{q}\right)}^{1/q}$ for q∈(0,∞), and the $\ell_{\infty }$ norm is provided by ${∥x∥}_{\infty }=\max_{1\leq i\leq p}\left|x_{i}\right|$. For a matrix $A=\left(a_{ij}\right)\in R^{p\times q}$, the following matrix norms are defined: ${∥A∥}_{1}=\max_{1\leq j\leq q}\sum_{i=1}^{p}\left|a_{ij}\right|$, ${∥A∥}_{\infty }=\max_{1\leq i\leq p}\sum_{j=1}^{q}\left|a_{ij}\right|$, ${∥A∥}_{\max}=\max_{i,j}\left|a_{ij}\right|$, and ${∥A∥}_{2}=\Lambda_{\max}{\left(A^{⊤}A\right)}^{1/2}$, where $\Lambda_{\min}\left(A\right)$ and $\Lambda_{\max}\left(A\right)$ denote the smallest and largest eigenvalues of A, respectively.*

## 2. Adaptive CoCoLasso for Error-Prone Models

### 2.1. Model Setting

Consider the high-dimensional linear regression model

(1) y=Xβ+ε,

where $y={\left(y_{1},\ldots ,y_{n}\right)}^{⊤}$ represents the *n*-dimensional response vector, $X={\left(X_{1},\ldots ,X_{n}\right)}^{⊤}\in R^{n\times p}$ denotes the fixed design matrix, $\beta ={\left(\beta_{1},\ldots ,\beta_{p}\right)}^{⊤}$ is the unknown *p*-dimensional regression coefficient vector, $\varepsilon ∼N(0,\sigma^{2}I_{n})$ is an *n*-dimensional error vector independent of X, and $I_{n}$ is the n×n identity matrix (the Gaussian distribution is assumed for simplicity in analysis. However, similar theoretical results hold under the sub-Gaussian assumption provided that the tail probability of ε decays exponentially). Measurement errors in the design matrix are common in various applications, leading to the observation of a corrupted covariate matrix $W\in R^{n\times p}$ rather than the true matrix X.

Two classical cases are associated with measurement errors in the design matrix X. For cases of additive errors, the observed covariates are represented as W=X+A, where the rows of the additive error matrix $A={\left(a_{ij}\right)}_{n\times p}$ are independently and identically distributed (i.i.d.) with a mean vector 0 and a covariance matrix $\Sigma_{a}$. For cases of multiplicative errors, the observed covariates follow W=X⊙M, where ⊙ denotes the Hadamard product and the rows of the multiplicative error matrix $M={\left(m_{ij}\right)}_{n\times p}$ have mean vector $\mu_{m}$ and covariance matrix $\Sigma_{m}$. Missing data can be treated as a special case of this model, where the entries of M are Bernoulli random variables with success probability $1-\pi_{j}$, representing the probability of observing the *j*-th covariate, and $\pi_{j}$ denotes the missingness rate for the *j*-th covariate. To ensure model identifiability, the covariance matrix $\Sigma_{a}$ (for additive errors) or the pair $\left(\mu_{m},\Sigma_{m}\right)$ (for multiplicative errors) is assumed to be known, as in [16,18].

### 2.2. Adaptive CoCoLasso

In high-dimensional settings where the dimensionality *p* exceeds the sample size *n*, the true coefficient vector β is often assumed to be sparse. Specifically, the support set $S=\{j:\beta_{j}\neq 0\}$, representing the indices of truly relevant predictors, has size s=|S| satisfying $s=o\left(\frac{n}{\log p}\right)$. This sparsity assumption ensures model identifiability by requiring that only a small subset of predictors are nonzero, i.e., s≪n. Let $S^{C}$ denote the complementary set of *S*. In the context of clean data, penalized least squares methods are widely employed for sparse estimation of the true coefficient vector $\beta ={\left(\beta_{1},\ldots ,\beta_{p}\right)}^{⊤}$ in high-dimensional linear models. The loss function depends on Σ and ρ, where $\Sigma =\frac{1}{n}X^{⊤}X$ represents the Gram matrix and $\rho =\frac{1}{n}X^{⊤}y$ denotes the marginal correlation vector of (X,y), respectively. When the covariate matrix is affected by errors, [15] proposed unbiased estimators $\hat{\Sigma }$ and $\tilde{\rho }$ to approximate the unobservable quantities Σ and ρ. Specifically, these estimators can be expressed as

(2) $${\hat{\Sigma }}_{add}=\frac{1}{n}W^{⊤}W-\Sigma_{a},\;{\tilde{\rho }}_{add}=\frac{1}{n}W^{⊤}y$$

for the additive error cases and

(3) $${\hat{\Sigma }}_{mult}=\frac{1}{n}W^{⊤}W⊘\left(\Sigma_{m}+\mu_{m}\mu_{m}^{⊤}\right),\;{\tilde{\rho }}_{mult}=\frac{1}{n}W^{⊤}y⊘\mu_{m}$$

for the multiplicative error cases, where ⊘ denotes element-wise division.

However, the unbiased surrogate $\hat{\Sigma }$ is generally not positive semi-definite in high-dimensional scenarios. Consequently, $\hat{\Sigma }$ may possess a negative eigenvalue, resulting in the term ${\hat{\beta }}^{⊤}\hat{\Sigma }\hat{\beta }$ lacking a lower bound and causing the loss function to lose convexity. To resolve this problem, the unbiased surrogate $\hat{\Sigma }$ is replaced by its nearest positive semi-definite projection matrix, defined as $\tilde{\Sigma }=\arg\min_{\Sigma \geq 0}{∥\Sigma -\hat{\Sigma }∥}_{\max}$, which can be efficiently solved using the alternating direction method of multipliers (ADMM). By definition and the triangle inequality, it follows that

(4) $$\begin{array}{cc} ∥\tilde{\Sigma }-\hat{\Sigma }{∥}_{\max} & \leq ∥\tilde{\Sigma }{-\Sigma ∥}_{\max}+∥\Sigma -\hat{\Sigma }{∥}_{\max}\leq 2{∥\Sigma -\hat{\Sigma }∥}_{\max}, \end{array}$$

indicating that $\tilde{\Sigma }$ serves as an approximation to Σ with accuracy comparable to that of the unbiased estimate $\hat{\Sigma }$.

Following the processing of $\tilde{\Sigma }$ and ρ, Ref. [16] introduced the CoCoLasso method, which employs the $\ell_{1}$ penalty for regularization. However, the $L_{1}$ penalty often selects overly large models to minimize prediction risk. Motivated by [19], we adopt a weighted $L_{1}$ penalty to develop a convex objective function, where the weights are determined by an initial estimator. Suppose $\tilde{\beta }={\left({\tilde{\beta }}_{1},\ldots ,{\tilde{\beta }}_{p}\right)}^{⊤}$ is an initial estimator of β, which provides preliminary estimates of the regression coefficients. Based on this initial estimator, we define the weight vector $\omega ={\left(\omega_{1},\ldots ,\omega_{p}\right)}^{⊤}$, where $\omega_{j}={\left|{\tilde{\beta }}_{j}\right|}^{-1}$ for j=1,…,p. These weights enable the penalty to adapt to the relative importance of each variable, assigning larger penalties to coefficients with smaller initial estimates and smaller penalties to coefficients with larger initial estimates. Specifically, the proposed Adaptive CoCoLasso estimator $\hat{\beta }$ is defined as the optimal solution to the following optimization problem, computed after obtaining $\tilde{\Sigma }$ and $\tilde{\rho }$ as provided in (2) and (3):

(5) $$\begin{array}{cc} \hat{\beta } & =\arg\min_{\beta \in R^{p}}\left\{\frac{1}{2}\beta^{⊤}\tilde{\Sigma }\beta -{\tilde{\rho }}^{⊤}\beta +2\lambda \sum_{j=1}^{p}\omega_{j}\left|\beta_{j}\right|\right\}, \end{array}$$

where $\lambda =c_{0}s{\left[\frac{\log p}{n}\right]}^{1/2}$ for some positive constant $c_{0}$. Let $n^{-1/2}\tilde{W}\in R^{p\times p}$ be the Cholesky factor of $\tilde{\Sigma }$, such that $n^{-1}{\tilde{W}}^{⊤}\tilde{W}=\tilde{\Sigma }$, and define $\tilde{y}\in R^{p}$ to satisfy $n^{-1}{\tilde{W}}^{⊤}\tilde{y}=\tilde{\rho }$. Then, the proposed Adaptive CoCoLasso estimator $\hat{\beta }$ defined in (5) can be reformulated equivalently as the global minimizer of the following optimization problem

(6) $$\begin{array}{cc} \hat{\beta } & =\arg\min_{\beta \in R^{p}}\left\{\frac{1}{2n}∥\tilde{y}-\tilde{W}{\beta ∥}_{2}^{2}+2\lambda \sum_{j=1}^{p}\omega_{j}\left|\beta_{j}\right|\right\}. \end{array}$$

For comparison, CoCoLasso solves the following optimization problem:

$${\hat{\beta }}^{\mathrm{CoCoLasso}}\;=\arg\min_{\beta \in R^{p}}\left\{\frac{1}{2n}∥\tilde{y}-\tilde{W}{\beta ∥}_{2}^{2}+\lambda \sum_{j=1}^{p}\left|\beta_{j}\right|\right\}.$$

Here, the penalty term $\lambda \sum_{j=1}^{p}\left|\beta_{j}\right|$ introduces sparsity by shrinking some coefficients to exactly zero, effectively performing variable selection. However, since the same penalty λ>0 is applied to all coefficients, it tends to introduce bias, particularly for larger coefficients. Unlike CoCoLasso, Adaptive CoCoLasso incorporates data-driven weights $\omega_{j}$, where $\omega_{j}={\left|{\tilde{\beta }}_{j}\right|}^{-1}$, into the penalty term $\sum_{j=1}^{p}\omega_{j}\left|\beta_{j}\right|,$ adjusting the penalty to reflect the relative importance of each variable. This weighting scheme enables the Adaptive CoCoLasso to handle cases where variables have vastly different scales or signal strengths. Assigning smaller penalties to variables with larger estimated coefficients avoids over-penalizing important predictors, improving both variable selection accuracy and coefficient estimation. Additionally, the Adaptive CoCoLasso enhances the recovery of weak signals and reduces bias introduced by uniform penalties in traditional CoCoLasso.

## 3. Theoretical Properties

In this section, we rigorously derive the statistical error bounds for the Adaptive CoCoLasso estimate under $\ell_{1}$ and $\ell_{2}$ norms and establish theoretical guarantees for exact support recovery with high probability. Before discussing the theoretical results, we outline four technical assumptions.

**Condition 1.**  *The distributions of $\hat{\Sigma }$ and $\tilde{\rho }$ are identified by a set of parameters θ. Then, there exist generic constants C and c and positive functions ξ and $\epsilon_{0}$ depending on $\beta_{S},\beta_{S^{C}}$, and $\sigma^{2}$ such that, for every $\epsilon \leq \epsilon_{0}$, $\hat{\Sigma }$ and $\tilde{\rho }$ satisfy the following probability statements:*

(7) $$\begin{array}{cc} P(|{\hat{\Sigma }}_{ij}-\Sigma_{ij}|\geq \epsilon ) & \leq C\exp(-cn\epsilon^{2}\xi^{-1}),\;for\;any\;i,j=1,\ldots ,p; \end{array}$$

(8) $$\begin{array}{cc} P(|{\tilde{\rho }}_{j}-\rho_{j}|\geq \epsilon ) & \leq C\exp(-cns^{-2}\epsilon^{2}\xi^{-1}),\;for\;any\;j=1,\ldots ,p. \end{array}$$

**Condition 2.**  *For some positive constant κ, assume*

$$0<\kappa =\min_{∥v_{S^{C}}{∥}_{1}\leq 3{∥v_{S}∥}_{1}}\frac{v^{⊤}\Sigma v}{{∥v∥}_{2}^{2}},$$

*where $v={\left(v_{S}^{⊤},v_{S^{C}}^{⊤}\right)}^{⊤}\in R^{p}$, with $v_{S}$ and $v_{S^{C}}$ representing the subvectors corresponding to the support set S and its complement $S^{C}$, respectively.*

**Condition 3.**  *The minimum signal strength satisfies $\min_{j\in S}\left|\beta_{j}\right|\geq C\sqrt{s}\lambda ,$ where C>0 is a positive constant.*

**Condition 4.**  *The initial estimator $\tilde{\beta }$ satisfies $∥\tilde{\beta }{-\beta ∥}_{2}=O_{P}\left(\sqrt{s}\lambda \right)$ with $\lambda =c_{0}s{\left[\frac{\log p}{n}\right]}^{1/2}$.*

Condition 1, known as the closeness condition proposed by [16], requires that surrogates $\hat{\Sigma }$ (and consequently $\tilde{\Sigma }$) and $\tilde{\rho }$ achieve sufficient element-wise closeness to Σ and ρ, respectively. This condition has already been proven in [16] for typical additive and multiplicative measurement error cases, with $\hat{\Sigma }$ and $\tilde{\rho }$ defined in Equations (2) and (3). Condition 2, the restricted eigenvalue (RE) condition, ensures the stability and non-degeneracy of the design matrix on sparse predictor subsets. A similar RE condition was used in [20] to derive statistical error bounds for the clean Lasso estimator. Condition 3 specifies the minimum signal strength, ensuring that the true signal is large enough relative to the regularization parameter λ to differentiate significant predictors from noise. This condition is commonly assumed in high-dimensional regression settings to guarantee consistent variable selection and accurate estimation [21,22].

Condition 4 ensures that the initial estimator $\tilde{\beta }$ approximates the true parameter β with an $\ell_{2}$ error rate of $O_{P}\left(s\sqrt{\frac{s\log p}{n}}\right)$, providing the accuracy needed to construct adaptive weights that capture the underlying sparsity and improve the efficiency of the Adaptive CoCoLasso estimator. For clean covariates, commonly used initial estimators include Lasso [10], which leverages sparsity in high-dimensional settings, and ridge regression [23], which addresses multicollinearity effectively. Another widely used approach is the marginal regression estimator, which achieves zero-consistency under a partial orthogonality condition and is derived by fitting univariate regressions for each predictor separately [19]. When faced with a design matrix with measurement errors, CoCoLasso can be used as the initial estimator as it is specifically designed for measurement error models. Alternatively, the estimator introduced in [15], which provides theoretical guarantees for high-dimensional regression with noisy or missing data, can act as an initial estimator.

**Theorem 1.**  *Under Conditions 1–4, the Adaptive CoCoLasso estimator $\hat{\beta }$ satisfies the following oracle inequalities with high probability $1-O(p^{-c_{1}})$ for some positive constant $c_{1}$:*

$$∥\hat{\beta }{-\beta ∥}_{2}=O\left(\sqrt{s}\lambda \right),\;{∥\hat{\beta }-\beta ∥}_{1}=O\left(s\lambda \right).$$

*Here, $\lambda =c_{0}s{\left[\frac{\log p}{n}\right]}^{1/2}$, and the constants hidden in the O(·) notation depend on the restricted eigenvalue constant κ, the noise variance $\sigma^{2}$, and the probabilistic bounds specified in Condition 1.*

*Moreover, with high probability $1-O(p^{-c_{2}})$, it holds that*

$$P\left(\mathrm{supp}\left(\hat{\beta }\right)=\mathrm{supp}\left(\beta \right)\right)\to 1\;\mathrm{as}\;n\to \infty ,$$

*where $\mathrm{supp}\left(\beta \right)=\left\{j:\beta_{j}\neq 0\right\}$ denotes the support set of β, representing the indices of its nonzero components. Similarly, $\mathrm{supp}(\hat{\beta })$ represents the support set of the Adaptive CoCoLasso estimator $\hat{\beta }$.*

Theorem 1 establishes the theoretical properties of the Adaptive CoCoLasso estimator under high-dimensional linear models with measurement errors. Specifically, it guarantees oracle inequalities for the estimation errors in both $\ell_{2}$ and $\ell_{1}$ norms, showing that the errors scale with $\sqrt{s}\lambda$ and sλ, respectively. The constants in these bounds depend on key factors such as the restricted eigenvalue condition, noise variance, and probabilistic bounds. Tail probability is influenced by measurement errors, as described by the component ζ in Condition 1. Additionally, the theorem guarantees consistent support recovery, meaning that the true set of relevant predictors can be identified with high probability as the sample size *n* and dimensionality *p* increase. All proofs are provided in Appendix A.

## 4. Numerical Studies

Within this section, we utilize synthetic datasets to evaluate the effectiveness of the Adaptive CoCoLasso (A-CoCoLasso) estimator on finite samples. The comparison includes various alternative estimators, such as CoCoLasso [16], balanced estimation with $L_{1}$ regularization and smoothly clipped absolute deviation (B-SCAD), balanced estimation with $L_{1}$ regularization and hard-thresholding (B-Hard) [18], and the standalone hard-thresholding method. The Adaptive CoCoLasso weights were computed using CoCoLasso regression coefficients. The CoCoLasso and Adaptive CoCoLasso estimators were implemented using the LARS algorithm. All the simulation studies were performed using R, focusing on both additive and multiplicative measurement errors to evaluate the performance of the proposed method. In all the numerical experiments, the penalty parameter λ was determined through 10-fold cross-validation.

To evaluate the aforementioned estimators, we employed the performance metrics introduced in [18]. The first two metrics are the count of correctly selected covariates (C) and the count of incorrectly selected covariates (IC). These are defined as $C=\mathrm{True}\;\mathrm{Positives}\;\;\left(\mathrm{TP}\right)=\sum_{j\in S}I\left({\hat{\beta }}_{j}\neq 0\right)$ and $IC=\mathrm{False}\;\mathrm{Positives}\;\left(\mathrm{FP}\right)=\sum_{j\notin S}I\left({\hat{\beta }}_{j}\neq 0\right)$, respectively. The third and fourth metrics are the prediction error (PE) and the mean squared error (MSE), provided by $\mathrm{PE}\left(\hat{\beta }\right)={\left(\beta -\hat{\beta }\right)}^{⊤}\Sigma_{X}\left(\beta -\hat{\beta }\right)$ and $\mathrm{MSE}\left(\hat{\beta }\right)={∥\beta -\hat{\beta }∥}_{2}^{2}$, respectively. These metrics collectively assess feature selection accuracy through C and IC while evaluating predictive performance and estimation performance via PE and MSE, thus providing a comprehensive comparison framework.

### 4.1. Additive Error Cases

**Example 1.**  *We followed the simulation setup in [18] and generated 100 datasets, each containing n=100 observations from the linear model y=Xβ+ε, with p=250, $\beta ={\left(3,1.5,0,0,2,0,\ldots ,0\right)}^{⊤}$, and σ=3. The components of X and ε were independently sampled from a multivariate normal distribution $N(0,\Sigma_{X})$. We considered two covariance structures for $\Sigma_{X}$: the autoregressive structure, where $\Sigma_{X}={\left(0.5^{\left|i-j\right|}\right)}_{1\leq i,j\leq p}$, and the compound symmetry structure, where $\Sigma_{X}=0.311^{⊤}+0.7I$. The contaminated covariates W were obtained as W=X+A, where the rows of A were independently drawn from $N(0,\tau^{2})$ with τ=0.75 and τ=1.25, respectively. The results for the five estimators are summarized in Table 1 and Table 2.*

The results in Table 1 and Table 2 highlight the comparative performance of the five methods under additive measurement errors in both the autoregressive and compound symmetric structures. Comparing A-CoCoLasso with CoCoLasso, A-CoCoLasso consistently achieved a higher number of C and fewer instances of IC. For example, in the autoregressive structure with τ=0.75, A-CoCoLasso attained C = 2.93, which is closer to the ideal value of 3, compared to CoCoLasso (C = 2.94) while significantly reducing IC from 12.54 (CoCoLasso) to 3.89. Furthermore, A-CoCoLasso demonstrated better prediction error (PE = 1.68) and mean squared error (MSE = 2.01) compared to CoCoLasso (PE = 3.65; MSE = 3.64). Similarly, when compared to Hard, A-CoCoLasso achieved a higher C (2.93 vs. 2.27) and better overall estimation and prediction performance. These results indicate that A-CoCoLasso provides more accurate variable selection and estimation than both CoCoLasso and Hard.

In comparison to the balanced estimation methods (B-SCAD and B-Hard), A-CoCoLasso also demonstrated superior performance, particularly in reducing IC while maintaining C closer to the ideal value. For example, in the compound symmetric structure with τ=1.25, A-CoCoLasso achieved C = 2.46, outperforming both B-SCAD (C = 2.07) and B-Hard (C = 1.64). Additionally, A-CoCoLasso maintained a competitive IC of 12.88, which is lower than that of B-SCAD (IC = 13.59), while achieving better prediction and estimation accuracy (PE = 6.80; MSE = 8.39) compared to both B-SCAD (PE = 8.01; MSE = 9.91) and B-Hard (PE = 8.60; MSE = 11.04). These results demonstrate that A-CoCoLasso not only balances the trade-off between correctly identifying covariates and excluding noise variables but also provides robust estimation and prediction accuracy under various settings with additive measurement errors.

**Example 2.**  *To investigate the performance of the methods under ultra-high-dimensional settings with additive measurement errors, we adopted a similar setting to that in [7]. The coefficient vector was specified as $\beta ={\left(1,-0.5,\;0.7,\;-1.2,\;-0.9,\;0.3,\;0.55,\;0,\;\ldots ,\;0\right)}^{⊤}$. The sample size, dimensionality, and noise level were set as n=80, p=1000, and σ=1, respectively, reflecting an ultra-high-dimensional setting. The variability in the additive errors was characterized by standard deviation values of τ=0.25 and 0.5. Table 3 summarizes the performance of the five methods under this setting.*

Table 3 presents the performance of the five methods under ultra-high-dimensional settings with additive measurement errors for τ=0.25 and τ=0.5. In terms of the number of correctly identified covariates (C), A-CoCoLasso achieved a competitive performance compared to CoCoLasso and B-SCAD, with values close to the ideal benchmark of 4 under τ=0.25 (C = 3.95), and slightly reduced performance under τ=0.5 (C = 3.56). In contrast, Hard demonstrated considerably lower values for C (3.14 for τ=0.25 and 2.36 for τ=0.5), indicating weaker variable selection ability.

When examining the number of incorrectly identified covariates (IC), A-CoCoLasso substantially outperformed CoCoLasso and B-SCAD, maintaining much lower IC values (12.7 for τ=0.25 and 11.05 for τ=0.5) compared to CoCoLasso (31.32 and 24.66, respectively) and B-SCAD (21.69 and 21.84, respectively). Hard achieved the smallest IC but at the cost of reduced C, highlighting its conservative nature. In terms of PE and MSE, A-CoCoLasso remained competitive, with PE = 0.90 and MSE = 1.55 for τ=0.25, and PE = 1.21 and MSE = 2.01 for τ=0.5, showing comparable or better results than Hard and B-Hard while maintaining a balanced variable selection performance.

### 4.2. Multiplicative Error Cases

**Example 3.**  *We evaluated the performance of Adaptive CoCoLasso and other competing methods, including CoCoLasso, Hard, and balanced estimation, under multiplicative measurement errors. The true model remained the same as in the additive error setup, as described in Example 1. To simulate the multiplicative errors, we generated W=X⊙M, where the components of $M={\left(m_{ij}\right)}_{n\times p}$ followed a log-normal distribution. Specifically, $\log(m_{ij})$ independently followed the same distribution as $N(0,\tau^{2})$, with τ=0.25 and τ=0.75. Table 4 and Table 5 present the outcomes for the multiplicative error scenarios.*

The results in Table 4 and Table 5 demonstrate that A-CoCoLasso exhibits strong performance under multiplicative measurement errors across both autoregressive and compound symmetric structures. Compared to the other methods, A-CoCoLasso achieved a desirable balance between correctly identifying covariates and maintaining low false discovery rates while also demonstrating competitive prediction and estimation accuracy. Its robustness under varying levels of multiplicative error (τ=0.25 and τ=0.75) further highlights its adaptability and effectiveness in handling challenging high-dimensional scenarios with multiplicative measurement errors.

**Example 4.**  *We examined the performance of Adaptive CoCoLasso in ultra-high-dimensional settings with multiplicative measurement errors. To maintain comparability with the additive error scenarios, the simulation setup remained largely consistent with that in Example 2, except that the standard deviation values of the multiplicative errors were specified as τ=0.1 and τ=0.2, ensuring a comparable signal-to-noise ratio. The performance of the five methods is summarized in Table 6.*

Table 6 presents the performance of five methods under ultra-high-dimensional settings with multiplicative measurement errors for τ=0.1 and τ=0.2. The results demonstrate that A-CoCoLasso achieved a desirable balance between correctly identifying covariates (C) and maintaining a low number of incorrectly identified covariates (IC) while delivering competitive prediction and estimation accuracy. For τ=0.1, A-CoCoLasso shows robust performance, with C = 4.18 and IC = 14.55, outperforming CoCoLasso in terms of IC (34.79) while maintaining similar predictive performance (PE = 0.78 vs. PE = 1.21 for CoCoLasso). As τ increased to 0.2, A-CoCoLasso remained effective with C = 3.57 and IC = 4.26, again demonstrating a significant reduction in IC compared to CoCoLasso (26.81). Additionally, A-CoCoLasso achieved comparable PE and MSE values to the best-performing methods, such as B-SCAD, while demonstrating better variable selection than Hard. Overall, these results highlight A-CoCoLasso’s ability to effectively balance variable selection and prediction accuracy under challenging ultra-high-dimensional multiplicative error settings.

## 5. Discussion

This paper introduces the Adaptive CoCoLasso estimator, designed to balance prediction accuracy and feature selection in high-dimensional linear regression with measurement errors, effectively addressing both additive and multiplicative cases. The proposed method combines two key techniques: the nearest positive semi-definite projection matrix for correcting measurement errors by reconstructing the covariate matrix and an adaptive weighted $\ell_{1}$ penalty, which enhances sparsity and variable selection by assigning data-driven weights to the coefficients. Unlike combined $\ell_{1}$ and concave regularization, which introduces computational challenges due to its non-convex nature and parameter tuning difficulties, Adaptive CoCoLasso retains the computational efficiency of convex optimization while providing robust estimation performance. The methodology leverages the LARS algorithm to solve the penalized optimization problem, ensuring scalability to high-dimensional settings. The theoretical analysis and simulation results show that the Adaptive CoCoLasso achieves robust prediction and estimation performance, effectively addressing overfitting and the challenges posed by contaminated data.

Future work could focus on extending the Adaptive CoCoLasso estimator to address statistical inference challenges, such as constructing confidence intervals and performing hypothesis testing. A major difficulty in these extensions arises from the unknown true covariate matrix, which not only makes predicting the response vector challenging but also hinders accurate noise level estimation, even with a reliable coefficient estimator. These issues fall outside the scope of this paper and represent intriguing directions for future research.

## Figures and Tables

**Table 1:** Means and standard errors (in parentheses) of four performance metrics for five methods under additive error cases over 100 replications in the autoregressive structure.

Measure	A-CoCoLasso	CoCoLasso	Hard	B-SCAD	B-Hard
τ=0.75					
C	2.93 (0.03)	2.94 (0.03)	2.27 (0.07)	2.85 (0.04)	2.69 (0.05)
IC	3.89 (0.40)	12.54 (0.75)	0.14 (0.04)	8.85 (0.53)	0.71 (0.18)
PE	1.68 (0.12)	3.65 (0.17)	3.01 (0.27)	2.82 (0.16)	2.26 (0.25)
MSE	2.01 (0.15)	3.64 (0.18)	3.67 (0.33)	3.06 (0.17)	2.58 (0.28)
τ=1.25					
C	2.79 (0.04)	2.72 (0.05)	1.78 (0.08)	2.55 (0.05)	2.14 (0.08)
IC	5.30 (0.60)	13.36 (0.86)	0.25 (0.06)	8.20 (0.47)	0.83 (0.24)
PE	5.40 (0.24)	8.69 (0.24)	6.51 (0.39)	7.69 (0.32)	6.45 (0.29)
MSE	5.01 (0.21)	7.53 (0.21)	6.11 (0.34)	6.60 (0.26)	5.79 (0.33)

**Table 2:** Means and standard errors (in parentheses) of four performance metrics for five methods under additive error cases over 100 replications in the compound symmetric structure.

Measure	A-CoCoLasso	CoCoLasso	Hard	B-SCAD	B-Hard
τ=0.75					
C	2.77 (0.04)	2.69 (0.05)	1.86 (0.08)	2.62 (0.05)	2.31 (0.07)
IC	8.37 (0.50)	14.95 (0.73)	0.45 (0.07)	8.88 (0.41)	1.89 (0.29)
PE	3.01 (0.15)	4.62 (0.19)	5.04 (0.41)	3.43 (0.20)	4.16 (0.31)
MSE	3.79 (0.20)	6.05 (0.25)	6.15 (0.49)	4.40 (0.25)	5.43 (0.42)
τ=1.25					
C	2.46 (0.06)	2.24 (0.07)	1.20 (0.07)	2.07 (0.07)	1.64 (0.07)
IC	12.88 (0.44)	18.84 (0.64)	1.24 (0.13)	13.59 (0.45)	3.64 (0.27)
PE	6.80 (0.21)	8.46 (0.22)	10.43 (0.52)	8.01 (0.30)	8.60 (0.31)
MSE	8.39 (0.26)	10.81 (0.28)	11.59 (0.62)	9.91 (0.36)	11.04 (0.44)

**Table 3:** Means and standard errors (in parentheses) of four performance metrics for five methods under additive error cases over 100 replications in the ultra-high-dimensional autoregressive structure.

Measure	A-CoCoLasso	CoCoLasso	Hard	B-SCAD	B-Hard
τ=0.25					
C	3.95 (0.07)	3.96 (0.07)	3.14(0.11)	4.30 (0.09)	3.88 (0.08)
IC	12.7 (1.48)	31.32 (2.03)	0.40 (0.07)	21.69 (1.52)	4.82 (1.49)
PE	0.90 (0.03)	1.37 (0.04)	0.95 (0.04)	0.82 (0.06)	0.88 (0.05)
MSE	1.55 (0.03)	2.19 (0.05)	1.70 (0.08)	1.45 (0.05)	1.52 (0.06)
τ=0.5					
C	3.56 (0.07)	3.54 (0.08)	2.36 (0.11)	3.69 (0.09)	3.49 (0.09)
IC	11.05 (1.26)	24.66 (1.32)	0.37 (0.10)	21.84(0.99)	5.05 (0.96)
PE	1.21 (0.03)	1.76 (0.04)	1.36 (0.06)	1.27 (0.04)	1.24 (0.05)
MSE	2.01 (0.04)	2.72 (0.04)	2.26 (0.08)	2.07 (0.06)	2.01 (0.06)

**Table 4:** Means and standard errors (in parentheses) of four performance metrics for five methods under multiplicative error cases over 100 replications in the autoregressive structure.

Measure	A-CoCoLasso	CoCoLasso	Hard	B-SCAD	B-Hard
τ=0.25					
C	3.00 (0.00)	3.00 (0.00)	2.67 (0.05)	2.99 (0.01)	2.69 (0.02)
IC	4.78 (0.58)	14.50 (0.93)	0.13 (0.05)	8.46 (0.55)	0.51 (0.06)
PE	0.70 (0.05)	1.90 (0.09)	1.02 (0.10)	0.99 (0.06)	0.65 (0.08)
MSE	0.82 (0.06)	1.82 (0.10)	1.41 (0.14)	1.07 (0.07)	0.77 (0.08)
τ=0.75					
C	2.90 (0.03)	2.85 (0.04)	2.11 (0.07)	2.79 (0.04)	2.56 (0.06)
IC	5.17 (0.50)	12.98(0.70)	0.27 (0.06)	9.10 (0.58)	0.88 (0.18)
PE	2.36 (0.15)	5.05 (0.20)	3.76 (0.25)	3.63 (0.21)	2.98 (0.23)
MSE	2.63 (0.16)	4.84 (0.18)	4.50 (0.31)	3.80 (0.20)	3.37 (0.26)

**Table 5:** Means and standard errors (in parentheses) of four performance metrics for five methods under multiplicative error cases over 100 replications in the compound symmetric structure.

Measure	A-CoCoLasso	CoCoLasso	Hard	B-SCAD	B-Hard
τ=0.25					
C	3.00 (0.00)	2.97 (0.02)	2.86 (0.03)	2.97 (0.02)	2.93 (0.03)
IC	8.11 (0.60)	14.79 (0.75)	0.16 (0.05)	8.69 (0.57)	2.03 (0.62)
PE	1.23 (0.07)	2.22 (0.10)	0.76 (0.09)	1.06 (0.06)	1.12 (0.13)
MSE	1.53 (0.09)	2.93 (0.14)	0.96 (0.12)	1.37 (0.08)	1.47 (0.18)
τ=0.75					
C	2.67 (0.05)	2.54 (0.06)	1.89 (0.07)	2.55 (0.05)	2.30 (0.06)
IC	9.88 (0.53)	15.76 (0.62)	0.57 (0.09)	10.79 (0.50)	2.77 (0.33)
PE	4.23 (0.18)	6.25 (0.22)	4.85 (0.33)	4.16 (0.21)	4.63 (0.28)
MSE	5.27 (0.24)	8.11 (0.29)	5.83 (0.41)	5.28 (0.27)	6.16 (0.38)

**Table 6:** Means and standard errors (in parentheses) of four performance metrics for five methods under multiplicative error cases over 100 replications in the ultra-high-dimensional autoregressive structure.

Measure	A-CoCoLasso	CoCoLasso	Hard	B-SCAD	B-Hard
τ=0.1					
C	4.18 (0.09)	4.22 (0.08)	3.51 (0.11)	4.56 (0.09)	4.03 (0.09)
IC	14.55 (1.55)	34.79 (3.78)	0.49 (0.11)	27.42 (3.70)	12.87 (4.12)
PE	0.78 (0.02)	1.21 (0.04)	0.83 (0.05)	0.76 (0.04)	0.82 (0.04)
MSE	1.37 (0.03)	1.99 (0.05)	1.50 (0.08)	1.35 (0.05)	1.44 (0.05)
τ=0.2					
C	3.57 (0.08)	4.00 (0.08)	3.21 (0.07)	4.29 (0.08)	3.92 (0.08)
IC	4.26 (1.55)	26.81 (1.45)	0.43 (0.09)	20.82 (0.94)	4.88 (1.09)
PE	0.92 (0.04)	1.28 (0.04)	0.93 (0.05)	0.81 (0.04)	0.87 (0.04)
MSE	1.60 (0.05)	2.10 (0.05)	1.66 (0.07)	1.44 (0.05)	1.50 (0.05)

## Data Availability

The original contributions presented in this study are included in the article. Further inquiries can be directed to author.

## Appendix A

### Appendix A.1. Proof of Theorem 1

**Proof.** Denote the estimation error by $\nu =\hat{\beta }-\beta$, where $\hat{\beta }$ is the global minimizer defined in the Adaptive CoCoLasso method. For simplicity of notation, we let $\lambda ∥\hat{\beta }{∥}_{1,\omega }$ denote $\lambda \sum_{j=1}^{p}\omega_{j}\left|\beta_{j}\right|$, where $∥\hat{\beta }{∥}_{1,\omega }=\sum_{j=1}^{p}\omega_{j}\left|\beta_{j}\right|$ represents the weighted $\ell_{1}$ norm. Based on the definition of $\hat{\beta }$ in (6), the following inequality holds

$$\frac{1}{2}{\hat{\beta }}^{⊤}\tilde{\Sigma }\hat{\beta }-{\tilde{\rho }}^{⊤}\hat{\beta }+2\lambda ∥\hat{\beta }{∥}_{1,\omega }\leq \frac{1}{2}\beta^{⊤}\tilde{\Sigma }\beta -{\tilde{\rho }}^{⊤}\beta +2\lambda {∥\beta ∥}_{1,\omega }.$$

By simple calculations and the definition of the $\ell_{\infty }$ norm, we have

(A1) $$\frac{1}{2}\nu^{⊤}\tilde{\Sigma }\nu +2\lambda ∥\hat{\beta }{∥}_{1,\omega }\leq {∥\nu ∥}_{1}∥\tilde{\rho }-\tilde{\Sigma }{\beta ∥}_{\infty }+2\lambda {∥\beta ∥}_{1,\omega }.$$

For better clarity, the proof will be divided into six steps, deriving bounds on the prediction based on the inequality above. **Step 1.** We derive a bound for the term $∥\tilde{\rho }-\tilde{\Sigma }{\beta ∥}_{\infty }$. By applying the triangle inequality, we obtain

(A2) $$∥\tilde{\rho }-\tilde{\Sigma }{\beta ∥}_{\infty }\leq ∥\tilde{\rho }{-\rho ∥}_{\infty }{+∥\rho -\Sigma \beta ∥}_{\infty }+{∥\left(\tilde{\Sigma }-\Sigma \right)\beta ∥}_{\infty }.$$

For the first part of the inequality (A2) on the right-hand side, $∥\tilde{\rho }{-\rho ∥}_{\infty }$, Condition 1 implies that

$$P(|{\tilde{\rho }}_{j}-\rho_{j}\left|\geq \lambda /6\right)\leq C\exp\left(-cns^{-2}\lambda^{2}\xi^{-1}\right),\;\mathrm{for}\;\mathrm{any}\;j=1,\ldots ,p.$$

Applying the union bound,

$$P\left(∥\tilde{\rho }{-\rho ∥}_{\infty }\geq \lambda /6\right)\leq \sum_{j=1}^{p}P\left(|{\tilde{\rho }}_{j}-\rho_{j}|\geq \lambda /6\right)\leq p\cdot C\exp\left(-cns^{-2}\lambda^{2}\xi^{-1}\right).$$

Thus, we have $∥\tilde{\rho }{-\rho ∥}_{\infty }=O_{P}\left(s\sqrt{\frac{\log p}{n}}\right).$ Next, we derive a bound for the second term ${∥\rho -\Sigma \beta ∥}_{\infty }$. Note that

$$\rho -\Sigma \beta =\frac{1}{n}X^{⊤}y-\Sigma \beta =\frac{1}{n}X^{⊤}\left(X\beta +\varepsilon \right)-\Sigma \beta =\frac{1}{n}X^{⊤}\varepsilon .$$

Under the assumption that $\varepsilon ∼N(0,\sigma^{2}I_{n})$, invoking Lemma A1, we have the probability bound

$$P\left({∥\rho -\Sigma \beta ∥}_{\infty }>\lambda /6\right)\leq p\cdot C\exp\left(-cn\lambda^{2}\sigma^{-2}\right),$$

for some constants C>0 and c>0. Therefore, with high probability, ${∥\rho -\Sigma \beta ∥}_{\infty }=O_{P}\left(\sqrt{\frac{\log p}{n}}\right).$ The third component $∥\left(\tilde{\Sigma }-\Sigma \right){\beta ∥}_{\infty }$ can be bounded as follows. Using Condition 1, for all i,j=1,…,p, we have

$$P\left(|{\tilde{\Sigma }}_{ij}-\Sigma_{ij}|\geq \epsilon \right)\leq C\exp\left(-cn\epsilon^{2}\xi^{-1}\right),$$

where C>0 and c>0 are constants. Combining Lemma A2, we have

$$∥\tilde{\Sigma }{-\Sigma ∥}_{\max}=O_{P}\left(\sqrt{\frac{\log p}{n}}\right).$$

For the sparse vector β with support size |S|=s, we additionally obtain

$$∥\left(\tilde{\Sigma }-\Sigma \right){\beta ∥}_{\infty }\leq ∥\tilde{\Sigma }{-\Sigma ∥}_{\max}{∥\beta ∥}_{1}.$$

Using ${∥\beta ∥}_{1}\leq \sqrt{s}{∥\beta ∥}_{2}$ and assuming ${∥\beta ∥}_{2}=O\left(1\right)$, it follows that

$$∥\left(\tilde{\Sigma }-\Sigma \right){\beta ∥}_{\infty }=O_{P}\left(s\sqrt{\frac{\log p}{n}}\right).$$

Combining the three parts above, we have

$$∥\tilde{\rho }-\tilde{\Sigma }{\beta ∥}_{\infty }\leq O_{P}\left(s\sqrt{\frac{\log p}{n}}\right)+O_{P}\left(\sqrt{\frac{\log p}{n}}\right)+O_{P}\left(s\sqrt{\frac{\log p}{n}}\right).$$

Since the sparsity *s* dominates in high-dimensional settings, the final bound is

(A3) $$∥\tilde{\rho }-\tilde{\Sigma }{\beta ∥}_{\infty }=O_{P}\left(s\sqrt{\frac{\log p}{n}}\right).$$

**Step 2.** Decompose the error term ν into components on the support set $S=\{j:\beta_{j}\neq 0\}$ and its complement $S^{C}$, so $\nu =\nu_{S}+\nu_{S^{C}}.$ The weighted $\ell_{1}$ norms can be written as

$$∥\hat{\beta }{∥}_{1,\omega }=∥{\hat{\beta }}_{S}{∥}_{1,\omega }+∥{\hat{\beta }}_{S^{C}}{∥}_{1,\omega }{,\;∥\beta ∥}_{1,\omega }={∥\beta_{S}∥}_{1,\omega }.$$

Substituting these into inequality (A1) yields

(A4) $$\frac{1}{2}\nu^{⊤}\tilde{\Sigma }\nu +2\lambda ∥{\hat{\beta }}_{S^{C}}{∥}_{1,\omega }\leq \nu^{⊤}\left(\tilde{\rho }-\tilde{\Sigma }\beta \right)+2\lambda {∥\nu_{S}∥}_{1,\omega }.$$

For the first term on the left-hand side of (A4), we have

$$\nu^{⊤}\tilde{\Sigma }\nu =\nu^{⊤}\Sigma \nu +\nu^{⊤}\Delta \nu ,$$

where $\Delta =\tilde{\Sigma }-\Sigma$ and $\Sigma =\frac{1}{n}X^{⊤}X$. By applying Condition 2 for any ν satisfying $∥\nu_{S^{C}}{∥}_{1}\leq 3{∥\nu_{S}∥}_{1}$, we have

$$\nu^{⊤}\Sigma \nu \geq n\kappa {∥\nu_{S}∥}_{2}^{2},$$

where κ>0 is the restricted eigenvalue constant. For the error term $\Delta =\tilde{\Sigma }-\Sigma$, Condition 1 ensures that

$${∥\Delta ∥}_{\max}\leq O_{P}\left(\sqrt{\frac{\log p}{n}}\right).$$

We can bound $|\nu^{⊤}\Delta \nu |$ as follows: $|\nu^{⊤}{\Delta \nu |\leq ∥\Delta ∥}_{\max}{∥\nu ∥}_{1}^{2}.$ From the sparsity assumption, ${∥\nu ∥}_{1}=∥\nu_{S}{∥}_{1}+∥\nu_{S^{C}}{∥}_{1}\leq 4{∥\nu_{S}∥}_{1}$. Applying the Cauchy–Schwarz inequality $∥\nu_{S}{∥}_{1}\leq \sqrt{s}{∥\nu_{S}∥}_{2},$ where s=|S|, the size of the support set, we obtain

$$|\nu^{⊤}\Delta \nu |\leq O_{P}\left(\sqrt{\frac{\log p}{n}}\right)\cdot (4\sqrt{s}∥\nu_{S}{{∥}_{2})}^{2}.$$

Combining the bounds for $\nu^{⊤}\Sigma \nu$ and $\nu^{⊤}\Delta \nu$, we have

$$\nu^{⊤}\tilde{\Sigma }\nu =\nu^{⊤}\Sigma \nu +\nu^{⊤}\Delta \nu \geq n\kappa ∥\nu_{S}{∥}_{2}^{2}-O_{P}\left(s\sqrt{\frac{\log p}{n}}\right){∥\nu_{S}∥}_{2}^{2}.$$

When $n\gg s\sqrt{\log p}$, the second term is asymptotically negligible. Redefining the restricted eigenvalue constant as $\kappa^{\prime }=\kappa /2>0$, we ensure that the first term on the left-hand side of (A4) satisfies $\nu^{⊤}\tilde{\Sigma }\nu \geq n\kappa^{\prime }{∥\nu_{S}∥}_{2}^{2}.$ Bound the first term $\nu^{⊤}\left(\tilde{\rho }-\tilde{\Sigma }\beta \right)$ on the right-hand side of (A4) and we have

$$\nu^{⊤}\left(\tilde{\rho }-\tilde{\Sigma }\beta \right)=\sum_{j\in S}\nu_{j}\left({\tilde{\rho }}_{j}-{\tilde{\Sigma }}_{j}^{⊤}\beta \right)+\sum_{j\notin S}\nu_{j}\left({\tilde{\rho }}_{j}-{\tilde{\Sigma }}_{j}^{⊤}\beta \right).$$

Using the bound in (A3) $∥\tilde{\rho }-\tilde{\Sigma }{\beta ∥}_{\infty }\leq O_{P}\left(s\sqrt{\frac{\log p}{n}}\right),$ we have

$$|\nu^{⊤}\left(\tilde{\rho }-\tilde{\Sigma }\beta \right){|\leq ∥\nu ∥}_{1}\cdot O_{P}\left(s\sqrt{\frac{\log p}{n}}\right).$$

For the sparsity structure of ν, ${∥\nu ∥}_{1}=∥\nu_{S}{∥}_{1}+{∥\nu_{S^{C}}∥}_{1}$. Under the sparsity constraint $∥\nu_{S^{C}}{∥}_{1}\leq 3{∥\nu_{S}∥}_{1}$, we obtain ${∥\nu ∥}_{1}\leq 4{∥\nu_{S}∥}_{1}.$ Further, by the Cauchy–Schwarz inequality $∥\nu_{S}{∥}_{1}\leq \sqrt{s}{∥\nu_{S}∥}_{2},$ we have

$$|\nu^{⊤}\left(\tilde{\rho }-\tilde{\Sigma }\beta \right)|\leq 4\sqrt{s}{∥\nu_{S}∥}_{2}\cdot O_{P}\left(s\sqrt{\frac{\log p}{n}}\right).$$

Simplifying, this yields

(A5) $$|\nu^{⊤}\left(\tilde{\rho }-\tilde{\Sigma }\beta \right)|\leq O_{P}\left(\sqrt{s}\lambda \right){∥\nu_{S}∥}_{2}.$$

Substituting bounds (A5) and

$$\nu^{⊤}\tilde{\Sigma }\nu \geq n\kappa^{\prime }{∥\nu_{S}∥}_{2}^{2}$$

into inequality (A4), we obtain

(A6) $$\frac{1}{2}n\kappa^{\prime }∥\nu_{S}{∥}_{2}^{2}+2\lambda ∥{\hat{\beta }}_{S^{C}}{∥}_{1,\omega }\leq O_{P}\left(\sqrt{s}\lambda \right)∥\nu_{S}{∥}_{2}+2\lambda {∥\nu_{S}∥}_{1,\omega }.$$

**Step 3.** To bound $∥{\hat{\beta }}_{S^{C}}{∥}_{1,\omega }$ on the left-hand side of inequality (A6), we observe that $∥{\hat{\beta }}_{S^{C}}{∥}_{1,\omega }=\sum_{j\in S^{C}}\omega_{j}\left|{\hat{\beta }}_{j}\right|,$ where the weights $\omega =(\omega_{1},\ldots ,\omega_{p})$ are defined based on the initial estimator $\tilde{\beta }$, with $\omega_{j}={\left|{\tilde{\beta }}_{j}\right|}^{-1}$ for j=1,…,p. Under Condition 4, the initial estimator satisfies $∥\tilde{\beta }{-\beta ∥}_{2}=O_{P}\left(\sqrt{s}\lambda \right)$, and the sparsity assumption implies that $\beta_{j}=0$ for $j\in S^{C}$. By the consistency of $\tilde{\beta }$, we have $|{\tilde{\beta }}_{j}|=O_{P}\left(\sqrt{s}\lambda \right)$ for $j\in S^{C}$, leading to $\omega_{j}=O_{P}\left(\frac{1}{\sqrt{s}\lambda }\right)$. Thus, the penalty term $2\lambda \omega_{j}\left|\beta_{j}\right|$ in the objective function (6) becomes $2\lambda \omega_{j}\left|\beta_{j}\right|=O_{P}\left(\lambda \cdot \omega_{j}\cdot \left|\beta_{j}\right|\right)=O_{P}\left(\frac{1}{\sqrt{s}}\left|\beta_{j}\right|\right).$ Under Condition 3, as $\lambda \omega_{j}$ becomes large for $j\in S^{C}$, the penalty ensures $P({\hat{\beta }}_{j}=0)\to 1$ as n→∞. To bound $∥\nu_{S}{∥}_{1,\omega }$ on the right-hand side of (A6), we note that the weighted $\ell_{1}$ norm satisfies $∥\nu_{S}{∥}_{1,\omega }=\sum_{j\in S}\omega_{j}\left|\nu_{j}\right|,$ where $\omega_{j}={\left|{\tilde{\beta }}_{j}\right|}^{-1}$, and ${\tilde{\beta }}_{j}$ denotes the initial estimator. Under Condition 4, the weights satisfy $|\omega_{j}|=O_{P}\left(1\right)$ for j∈S since ${\tilde{\beta }}_{j}$ is consistent and $\beta_{j}\neq 0$. Thus, the weighted $\ell_{1}$ norm satisfies $∥\nu_{S}{∥}_{1,\omega }=\sum_{j\in S}\omega_{j}|\nu_{j}|\leq C∥\nu_{S}{∥}_{1}$ for some constant C>0. Substituting, we obtain

(A7) $$\frac{1}{2}n\kappa^{\prime }∥\nu_{S}{∥}_{2}^{2}\leq ∥\nu_{S}{∥}_{1}\cdot O_{P}\left(s\sqrt{\frac{\log p}{n}}\right)+2C\lambda {∥\nu_{S}∥}_{1}.$$

**Step 4.** We derive the $\ell_{2}$ norm bound for the estimation error $\nu =\hat{\beta }-\beta$. Using the Cauchy–Schwarz inequality, the $\ell_{1}$ norm and $\ell_{2}$ norm satisfy $∥\nu_{S}{∥}_{1}\leq \sqrt{s}{∥\nu_{S}∥}_{2}$, where s=|S| is the sparsity level. Substituting this, inequality (A7) can be rewritten as

$$\frac{1}{2}n\kappa^{\prime }∥\nu_{S}{∥}_{2}^{2}\leq \sqrt{s}∥\nu_{S}{∥}_{2}\cdot O_{P}\left(s\sqrt{\frac{\log p}{n}}\right)+2C\lambda \sqrt{s}{∥\nu_{S}∥}_{2}.$$

Factoring out $∥\nu_{S}{∥}_{2}$, we have

$$∥\nu_{S}{∥}_{2}\left(\frac{1}{2}n\kappa^{\prime }{∥\nu_{S}∥}_{2}-\sqrt{s}O_{P}\left(s\sqrt{\frac{\log p}{n}}\right)-2C\lambda \sqrt{s}\right)\leq 0.$$

For $∥\nu_{S}{∥}_{2}>0$, the term in parentheses must be non-positive. We have

$$∥\nu_{S}{∥}_{2}\leq \frac{2\sqrt{s}}{n\kappa^{\prime }}O_{P}\left(s\sqrt{\frac{\log p}{n}}\right)+\frac{4C\sqrt{s}\lambda }{n\kappa^{\prime }}.$$

Substituting $\lambda =O\left(s\sqrt{\frac{\log p}{n}}\right)$, we obtain $∥\nu_{S}{∥}_{2}\leq \frac{\sqrt{s}}{n\kappa^{\prime }}O_{P}\left(s\sqrt{\frac{\log p}{n}}\right).$ Simplifying, the $\ell_{2}$ norm of the error satisfies $∥\nu_{S}{∥}_{2}\leq C_{1}s\sqrt{\frac{s\log p}{n}},$ where $C_{1}>0$ is a constant depending on $\kappa^{\prime }$. Hence, we obtain

(A8) $$∥\hat{\beta }{-\beta ∥}_{2}={∥\nu_{S}∥}_{2}\leq C_{1}\sqrt{s}\lambda .$$

where $\lambda =s\sqrt{\frac{\log p}{n}}$ and $C_{1}$ is some positive constant. **Step 5.** We derive the $\ell_{1}$ norm bound for the estimation error $\nu =\hat{\beta }-\beta$. The $\ell_{1}$ norm of ν decomposes as ${∥\nu ∥}_{1}=∥\nu_{S}{∥}_{1}+{∥\nu_{S^{C}}∥}_{1}$. By the sparsity assumption, $∥\nu_{S^{C}}{∥}_{1}\leq 3{∥\nu_{S}∥}_{1}$, we have ${∥\nu ∥}_{1}\leq 4{∥\nu_{S}∥}_{1}.$ By the Cauchy–Schwarz inequality, the $\ell_{1}$ norm on the support set *S* satisfies $∥\nu_{S}{∥}_{1}\leq \sqrt{s}{∥\nu_{S}∥}_{2}$, where s=|S| is the sparsity level. Substituting the $\ell_{2}$ norm bound from inequality (A8), $∥\nu_{S}{∥}_{2}\leq C_{1}\sqrt{s\frac{s\log p}{n}}$, where $C_{1}>0$ depends on $\kappa^{\prime }$, *n*, and *p*. Substituting into the inequality for $∥\nu_{S}{∥}_{1}$, we have $∥\nu_{S}{∥}_{1}\leq s\sqrt{s}\cdot C_{1}\sqrt{\frac{s\log p}{n}}=C_{1}\frac{s^{2}\log p}{n}$. Combining the bounds for $\nu_{S}$ and $\nu_{S^{C}}$, the $\ell_{1}$ norm satisfies ${∥\nu ∥}_{1}\leq 4{∥\nu_{S}∥}_{1}\leq 4C_{1}\frac{s^{2}\log p}{n}$. Defining $C_{2}=4C_{1}$, we conclude

$$∥\hat{\beta }{-\beta ∥}_{1}={∥\nu ∥}_{1}\leq C_{2}s\lambda ,$$

where $\lambda =s\sqrt{\frac{\log p}{n}}$, $C_{2}>0$ depends on $\kappa^{\prime }$, *s*, *n*, and *p*. **Step 6.** We aim to show that $\hat{\beta }$ correctly identifies the support set of the true regression coefficients β, such that $P\left(\mathrm{supp}\left(\hat{\beta }\right)=\mathrm{supp}\left(\beta \right)\right)\to 1\;\mathrm{as}\;n\to \infty .$ For a vector $\nu =\hat{\beta }-\beta$, we have ${∥\nu ∥}_{\infty }\leq {∥\nu ∥}_{2}.$ From inequality (A8), the $\ell_{2}$ norm of the error satisfies $∥\hat{\beta }{-\beta ∥}_{2}\leq C_{1}\sqrt{s}\lambda .$ Then, we conclude that $∥\hat{\beta }{-\beta ∥}_{\infty }\leq O_{P}\left(\sqrt{s}\lambda \right).$ For each j∈S, the minimum signal strength, Condition 3 implies $|\beta_{j}|\geq C\sqrt{s}\lambda .$ With high probability, the estimation error satisfies $|{\hat{\beta }}_{j}-\beta_{j}|\leq O_{P}\left(\sqrt{s}\lambda \right).$ For j∈S, combining this with the minimum signal strength, Condition 3 provides $|{\hat{\beta }}_{j}|\geq Cc_{0}\sqrt{s}\lambda -O_{P}\left(\sqrt{s}\lambda \right).$ For sufficiently large *n*, where $Cc_{0}>1$, this ensures $|{\hat{\beta }}_{j}|>0$. For j∉supp(β), the estimation error simplifies to $|{\hat{\beta }}_{j}\left|=\right|{\hat{\beta }}_{j}-\beta_{j}\left|=\right|{\hat{\beta }}_{j}|$. The $\ell_{\infty }$ norm error bound satisfies $∥\hat{\beta }{-\beta ∥}_{\infty }\leq O_{P}\left(\sqrt{s}\lambda \right)$, which implies $|{\hat{\beta }}_{j}|\leq O_{P}\left(\sqrt{s}\lambda \right)$. In addition, by Condition 3, there is $|{\hat{\beta }}_{j}|\geq C\sqrt{s}\lambda$. This implies $O_{P}\left(\sqrt{s}\lambda \right)\geq \left|{\hat{\beta }}_{j}\right|\geq C\sqrt{s}\lambda$. This leads to a contradiction. Therefore, ${\hat{\beta }}_{j}=0$ for j∉supp(β). Combining the cases for j∈S and j∉S, we conclude that $P\left(\mathrm{supp}\left(\hat{\beta }\right)=\mathrm{supp}\left(\beta \right)\right)\to 1\;\mathrm{as}\;n\to \infty .$ □

### Appendix A.2. Proof of Lemmas

**Lemma A1.** 
*Let $X\in R^{n\times p}$ denote a fixed design matrix and $\varepsilon ∼N(0,\sigma^{2}I_{n})$ represent an n-dimensional error vector, where $I_{n}$ is the n×n identity matrix. For any t>0, we define $z=\sigma \sqrt{\frac{t^{2}+2\log p}{n}}$. Under these settings, the following inequality holds*

$$P(∥n^{-1}X^{⊤}\varepsilon {∥}_{\infty }\leq z)\geq 1-2\exp\left\{-t^{2}/2\right\}.$$

**Proof.** Given that $\varepsilon ∼N(0,\sigma^{2}I_{n})$, for t>0 and $z=\sigma \sqrt{\frac{t^{2}+2\log p}{n}}$, we have

$$1-P(∥n^{-1}X^{⊤}{\varepsilon ∥}_{\infty }\leq z)=P(∥n^{-1}X^{⊤}\varepsilon {∥}_{\infty }>z)=P\left(\max_{1\leq j\leq p}\frac{|\varepsilon^{⊤}X_{j}|}{n}>z\right),$$

where $X_{j}$ is the *j*-th column of X. Using the union bound,

$$P\left(\max_{1\leq j\leq p}\frac{|\varepsilon^{⊤}X_{j}|}{n}>z\right)\leq \sum_{j=1}^{p}P\left(\frac{|\varepsilon^{⊤}X_{j}|}{\sqrt{n}\sigma }>\sqrt{t^{2}+2\log p}\right).$$

Since $\frac{\varepsilon^{⊤}X_{j}}{\sqrt{n}\sigma }∼N\left(0,1\right)$,

$$P\left(\frac{|\varepsilon^{⊤}X_{j}|}{\sqrt{n}\sigma }>\sqrt{t^{2}+2\log p}\right)=2P\left(\frac{\varepsilon^{⊤}X_{j}}{\sqrt{n}\sigma }>\sqrt{t^{2}+2\log p}\right).$$

Thus,

$$P\left(\max_{1\leq j\leq p}\frac{|\varepsilon^{⊤}X_{j}|}{n}>z\right)\leq 2p\exp\left\{-\left(\frac{t^{2}}{2}+\log p\right)\right\}.$$

Simplifying,

$$P(∥n^{-1}X^{⊤}\varepsilon {∥}_{\infty }>z)\leq 2\exp\left(-\frac{t^{2}}{2}\right).$$

The proof of Lemma A1 is now complete.□

**Lemma A2.** 
*For any ϵ>0, $P(∥\tilde{\Sigma }{-\Sigma ∥}_{\max}\geq \epsilon )\leq p^{2}\max_{i,j}P(|{\hat{\Sigma }}_{i,j}-\Sigma_{i,j}\left|\geq \epsilon /2\right).$*

**Proof.** From the inequality

$$∥\tilde{\Sigma }{-\Sigma ∥}_{\max}\leq ∥\tilde{\Sigma }-\hat{\Sigma }{∥}_{\max}+∥\hat{\Sigma }{-\Sigma ∥}_{\max}\leq 2{∥\hat{\Sigma }-\Sigma ∥}_{\max},$$

it follows that $P(∥\tilde{\Sigma }{-\Sigma ∥}_{\max}\geq \epsilon )\leq P(∥\hat{\Sigma }-\Sigma {∥}_{\max}\geq \epsilon /2).$ The result is obtained by applying the union bound over $P(|{\hat{\Sigma }}_{i,j}-\Sigma_{i,j}|\geq \epsilon /2)$ for all i,j. □
